# Correction to: New persistent plant RNA virus carries mutations to weaken viral suppression of antiviral RNA interference

**DOI:** 10.1111/mpp.70035

**Published:** 2024-12-10

## Abstract

Zhu, L.‐J., Zhu, Y., Zou, C., Su, L.‐Y., Zhang, C.‐T., Wang, C. et al. (2024) New persistent plant RNA virus carries mutations to weaken viral suppression of antiviral RNA interference. Molecular Plant Pathology, 25, e70020. Available from: https://doi.org/10.1111/mpp.70020

The following errors have been identified in the above published article:

1. The co‐first authors and co‐corresponding authors are not indicated.
2. The order of the funds appearing in the Funding information of the article are not consistent with the order in the Acknowledgements.

The authors would like to correct these errors as follows:

1. Li‐Juan Zhu and Yu Zhu contributed equally to this work.
  Jian‐Guo Wu and Yan‐Hong Han are co‐corresponding authors.
  Jiang‐Guo Wu: wujianguo81@126.com; Yan‐Hong Han: yan-hong@fafu.edu
2. The correct order of funds is: National Natural Science Foundation of China, Grant/Award Number 32025031 and 31,900,153, National Key Research and Development Program of China, Grant/Award Number 2023YFF1000500 and Special Fund Project for Science and Technology Innovation of FAFU, Grant/Award Number KFB23013.

We apologise for these errors.

Zhu, L.‐J., Zhu, Y., Zou, C., Su, L.‐Y., Zhang, C.‐T., Wang, C. et al. (2024) New persistent plant RNA virus carries mutations to weaken viral suppression of antiviral RNA interference. Molecular Plant Pathology, 25, e70020. Available from: https://doi.org/10.1111/mpp.70020

The following errors have been identified in the above published article:

1. The co‐first authors and co‐corresponding authors are not indicated.
2. The order of the funds appearing in the Funding information of the article are not consistent with the order in the Acknowledgements.

The authors would like to correct these errors as follows:

1. Li‐Juan Zhu and Yu Zhu contributed equally to this work.
  Jian‐Guo Wu and Yan‐Hong Han are co‐corresponding authors.
  Jiang‐Guo Wu: wujianguo81@126.com; Yan‐Hong Han: yan-hong@fafu.edu
2. The correct order of funds is: National Natural Science Foundation of China, Grant/Award Number 32025031 and 31,900,153, National Key Research and Development Program of China, Grant/Award Number 2023YFF1000500 and Special Fund Project for Science and Technology Innovation of FAFU, Grant/Award Number KFB23013.

We apologise for these errors.

